# Supplementary material for: Powerful and accurate detection of temporal gene expression patterns from multi-sample multi-stage single-cell transcriptomics data with TDEseq
Source: Genome Biol. 2024 Apr 15;25:96. doi: 10.1186/s13059-024-03237-3 (PMC11020788; doi:10.1186/s13059-024-03237-3)
Supplement: Supplementary file 4 — Additional file 4. Review history. [file 13059_2024_3237_MOESM4_ESM.docx]

Review History

**First round of review**

**Reviewer 1**

**Were you able to assess all statistics in the manuscript, including the appropriateness of statistical tests used?** Yes: I was able to assess all the results presented, but not the appropriateness of the fitting procedures underlying the method itself.

**Were you able to directly test the methods?** Yes.

**Comments to author:**

The paper presents a method using smoothing spline functions and linear additive mixed model (LAMM) to detect temporal patterns (in particular, growths, recessions, peaks and troughs) of gene expression in single-cell data. The paper includes a description of the method (involving what seems to be, from the little I could actually understand of them, relatively fancy fitting tricks), a validation on simulations, and applications to real datasets. The method attempts to solve a relevant problem, the package is relatively well documented, and the paper is generally well written (though some clarififcations would help).

For reasons indicated below, I am not yet entirely convinced by its claim of superiority to alternatives, and there are some important issues that I believe should be addressed before publications.

MAJOR ISSUES

1. FRAMING OF THE PROBLEM

The first and most fundamental point is an important distinction between two types of temporal scRNA data which it would be critical to discuss in the paper. First, there are datasets in which nearly all cells in a stage will show the stage-specific expression (or would show it if they were accurately quantified), as seems to be the case in the lung cancer dataset. However, there are also datasets where the cells progress at different rates, so that at the second experimental timepoint, for example, some cells are still transcriptionally like at the first, and others are already like the third, or halfway there. This is typical of differentiation datasets, and (from looking at the heatmaps) seems to be the case in the liver development dataset. For simplicity, I'll called them 'smudged' datasets. Methods like tradeseq, which work using the cells' pseudotime, are inherently designed to capture the extra information afforded by cells that might be in-between experimental timepoints. Instead, methods like TDESeq assign a cell its sample's timepoint, not using such information. This might not at all be a problem in some datasets, but must be made very clear to the readers.

2. SIMULATIONS

This first point, however leads me to a problem with the simulations: namely that they are designed to generate distinct stages, rather than smudged datasets, making it an unfair comparison. Again, there is no problem in measuring the performance of the methods on this task, so long as it is clear what the task is and is not, and, ideally, a similar simulation is done to compare the methods on the smudged task.

Regarding the simulations, a few things should additionally be clarified. First, it's unclear whether the authors simulated only one sample per timepoint, or with replication. In addition, it would be useful for users not familiar with Splatter to explain what a temporal effect size of 0.4 means, roughly.

Second, from the paper it's not clear how the temporal patterns are simulated. The authors only indicate that the changes are made from a baseline sample, and how the changes are passed to Splatter, but not how the changes are related across timepoints. This should be more clearly described.

Since the power and FDR results presented are split by temporal pattern, I assume that the authors simulate explicitly the temporal patterns explicitly tested by their own model, which obviously represents a certain bias. In some contexts, especially with single-nuclei data (in which transcripts do not 'accumulate' over extended periods of time), some temporal transitions are bound to be happening between (rather than across) the timepoints, i.e. for instance having a plateau in the first few timepoints, then another plateau in the last few timepoints, without a clear gradation in-between (a 'tipping-point' change, as the authors mention in the discussion). When comparing to methods that are agnostic as to a time-response pattern, it would therefore be important to demonstrate that TDEseq's gain in power to the 4 temporal patterns modeled is not accompanied by a loss of power for other types of changes which, though different from the ones modeled, are nonetheless relevant temporal patterns. These include the two plateaus just mentioned, as well as an upregulation at a single timepoint (e.g. in differentiation datasets it's very common to have a set of genes that are different in the very first timepoint, and then stable in the others). Even if TDEseq implied a loss of power in detecting such pattern it wouldn't be a tragedy, but it would need to be discussed explicitly, in order for users to be aware of it.

3. COMPARISONS

This brings me to the comparisons. While trade-seq and impulseDE2 are relevant comparisons, there are some other options. First, the psupertime R package is supposed to detect temporal patterns related to pseudotime while simultaneously using known sample-level stages/timepoints, thus in principle being adequate for both smudged and distinct scenarios.

Second, a number of reports (e.g. 10.1038/s41467-020-19894-4 ) indicate that pseudobulk differential expression using classical packages (e.g. edgeR) is equivalent or superior to many cell-level methods. In light of this, it would be important to compare, in the context of a simulation with replicated/multi-sample timepoints, TDEseq to a pseudobulk analysis taking the timepoints as categorical and testing dropping all of the time-related coefficients simultaneously (e.g. ~1+stage vs ~1, eventually including other relevant covariates), as is typically done in bulk RNAseq time-course analysis. This is especially important given TDEseq's main use-case being the distinct stages scenario discussed above.

4. PACKAGE

- The package depends on the Matrix.utils package, which was removed from CRAN a year ago. As a result, the installation fails on any decently-recent setup, and I had to find an older machine to test it. This should be solved before publication.

- I've tested the method on a dataset of ~5k cells and 15k genes, and it took a day to finish. I understand that the package has a pseudocell capability to reduce computational load, but since the fits are independent from each other, I don't understand why no multi-threading was implemented - it's really simple to do, and makes a big difference to users.

- In the documentation, the usage of the '@' (e.g. seurat_object@slot) to access data is not good practice and should be replaced by the accessor functions (e.g. GetAssayData and the likes). Also, in the help of the main function, the input should be more clearly defined, e.g. that 'meta' should have columns with certain names and classes (e.g. 'stage' can't be a factor but needs to be numeric).

MINOR POINTS:

- I think the average reader won't understand, from the intro, the distinction between time-resolved and time-course data. It might also not be necessary for the purpose of the paper.

- Some details in the text could be added to improve clarity, for example in the methods section, when the formula is discussed in more detailed, it would be good to spell out all variables (I had to scroll back and forth between results and methods to figure out what the variables are).

- In Supplementary Figure 10C, the plot doesn't at first glance convey the point made in the text (high correlation). It would be easier to interpret if it was colored by density, so that we'd see that the points not along the diagonal are a small minority. In addition, however, there's clearly a set of genes that get a much stronger linear p-value than mixed, and it would be worth discussing this briefly.

- Check for typos (e.g. p.9 line 16, distinct)

**Reviewer 2**

**Were you able to assess all statistics in the manuscript, including the appropriateness of statistical tests used?** No.

**Were you able to directly test the methods?** Yes.

**Comments to author:**

This research paper introduces a method, TDEseq, which offers a non-parametric approach for detecting temporal expression patterns across multiple time points. Below, I've listed my questions and suggestions:

1) In the introduction, the author mentioned that 'time is often treated as a categorical variable, and differential expression analysis is performed through pairwise comparisons using methods such as the two-sided Wilcoxon rank-sum test, DESeq2, or edgeR.' This statement should be refined. For example, when using edgeR, continuous variables are acceptable for constructing the model matrix.

2) The color of the Tradseq legend in the supplementary figures appears to be incorrect.

3) In the method section, it is mentioned that 'transformed genes' are used in the model to facilitate preprocessing before temporal expression gene detection. However, the term 'transformed' is not clearly explained. Is any normalization or integration method manipulation permitted? If so, then raw counts should also be allowed as well?

4) Could you provide details on what the power looks like in simulation scenarios where the effect size varies?

5) Based on my observations, in simulations, the mixed model seems better at detecting 'growth' and 'recession,' whereas the linear model appears better at capturing the 'peak' and 'trough.' Do you have any insights into this observation?

6) The term 'batch' is not well explained in the context of simulations. From my understanding, the author simulates scenarios with varying settings for each sample. I noticed that the mixed TDEseq consistently produces well-controlled p-values and exhibits good power compared to the linear model. I believe this is due to a well-modeled relationship between expression levels. However, it may be more appropriate to avoid referring to this as a 'batch effect.'?

7) In the discussion section, some additional details are needed following the statement 'Two versions of TDEseq were developed to accommodate different real data application scenarios.'

The results of applying these new methods to real data provide valuable insights.

**Response to Comments from Reviewers:**

-------------------
**Reviewer: 1#**

**General Comments:**

The paper presents a method using smoothing spline functions and linear additive mixed model (LAMM) to detect temporal patterns (in particular, growths, recessions, peaks and troughs) of gene expression in single-cell data. The paper includes a description of the method (involving what seems to be, from the little I could actually understand of them, relatively fancy fitting tricks), a validation on simulations, and applications to real datasets. The method attempts to solve a relevant problem, the package is relatively well documented, and the paper is generally well written (though some clarifications would help). For reasons indicated below, I am not yet entirely convinced by its claim of superiority to alternatives, and there are some important issues that I believe should be addressed before publications.

**Response**: Thanks for your thoughtful and detailed feedback on our paper. We appreciate your acknowledgment of the relevant problem we aim to address and your positive comments on the paper's overall quality, documentation, and writing. We take your concerns seriously and are committed to addressing them comprehensively. Our detailed responses to your specific comments are as follows:

**Specific Comments:**

1. FRAMING OF THE PROBLEM: The first and most fundamental point is an important distinction between two types of temporal scRNA data which it would be critical to discuss in the paper. First, there are datasets in which nearly all cells in a stage will show the stage-specific expression (or would show it if they were accurately quantified), as seems to be the case in the lung cancer dataset. However, there are also datasets where the cells progress at different rates, so that at the second experimental timepoint, for example, some cells are still transcriptionally like at the first, and others are already like the third, or halfway there. This is typical of differentiation datasets, and (from looking at the heatmaps) seems to be the case in the liver development dataset. For simplicity, I'll called them 'smudged' datasets. Methods like tradeseq, which work using the cells' pseudotime, are inherently designed to capture the extra information afforded by cells that might be in-between experimental timepoints. Instead, methods like TDESeq assign a cell its sample's timepoint, not using such information. This might not at all be a problem in some datasets, but must be made very clear to the readers.

**Response:** We appreciate your keen observation and acknowledge the importance of discussing this critical aspect in our paper. You rightly highlight the difference between datasets where nearly all cells in a stage exhibit stage-specific expression, as opposed to datasets where cells progress at different rates, resulting in a "smudged" pattern across experimental time points, commonly seen in differentiation datasets. To recognize the significance of clarifying this distinction for our readers, particularly in understanding the applicability of TDEseq in different types of datasets, we address this concern as follows:

(1) Simulations. We created an additional simulation data set that displays a "smudged" pattern across time points (Page 28, lines 23-36). Then, we performed all methods in comparison. As a result, with a high proportion of interwined cells, we found the pseudotime-based methods (i.e., tradeSeq or ImpulseDE2) were more powerful than TDEseq in terms of temporal expression gene detection (Figure S12). We presented these results in a new section (Page 10, lines 26-36 and Page 11, lines 1-11), and discussed these drawbacks of TDEseq in Disscussion section (Page 21, lines 20-21). The detailed responses for this question can be found in the second question, see below.
(2) Real data applications. We conducted scRNA-seq analysis on cell differentiation, specifically focusing on the hepatoblast-to-hepatocyte transition study, which corresponds to the second real data application in the manuscript. With the pseudotime as inputs, we observed both versions of TDEseq generated comparable results with tradeSeq and ImpulseDE2 for temporal expression gene detection (Figures S14G-S14I), presumably due to small proportion of interwined cells among time points. We presented these results in main text (Page 15, lines 19-24).
(3) Discussion. We ensured to incorporate a thorough discussion on these points in the revised manuscript (Page 21, lines 20-21, lines 32-36; Page 22, lines 1-4, lines 31-35; Page 23, lines 1-4), explicitly highlighting the scenarios where TDEseq may perform optimally and where the inherent design might have limitations, especially in "smudged" datasets.

We hope these revisions address your concerns and contribute to the overall enhancement of our manuscript. Thank you again for your valuable input.

2. SIMULATIONS
   This first point, however leads me to a problem with the simulations: namely that they are designed to generate distinct stages, rather than smudged datasets, making it an unfair comparison. Again, there is no problem in measuring the performance of the methods on this task, so long as it is clear what the task is and is not, and, ideally, a similar simulation is done to compare the methods on the smudged task.

**Response:** Thank you for bringing up this important point. In response to your comment, we conducted additional simulations to assess the performance of TDEseq in handling "smudged" datasets. To achieve this, we employed *Symsim* (R package for simulation of scRNA-Seq data; Page 28, lines 23-36 and Page 29, line 1) to generate different proportion of intertwined cells scRNA-seq data, and then Slingshot was utilized as the pseudotime inference. As TDEseq only requires numerical vectors for time point information, but it can also accommodate pseudotime as inputs.

In our new simulations, we demonstrate that TDEseq can detect temporal expression patterns with the four specified categories even in "smudged" datasets, although the power is lower compared to tradeSeq (Figure S12). Meanwhile, with increasing proportion of intertwined cells, tradeSeq was more powerful than TDEseq in temporal expression gene detection (Figure S12D). Besides, by taking pseudotime as time points, the power of TDEseq is comparable with tradeSeq and ImpulseDE2, and TDEseq can detect most of pattern-specific temporal expression genes (Figures S12E and S12F). The details of simulations were demonstrated in Materials and Methods section (Page 28, lines 23-36 and Page 29, line 1). The corresponding simulation results were shown in Results section (Page 10, lines 26-36 and Page 11, lines 1-11). We also discussed these drawbacks of TDEseq in Disscussion section (Page 21, lines 20-21).

We appreciate your valuable feedback, and this additional analysis has allowed us to provide a more comprehensive evaluation of the performance of TDEseq in scenarios involving "smudged" data sets.

3. Regarding the simulations, a few things should additionally be clarified. First, it's unclear whether the authors simulated only one sample per timepoint, or with replication. In addition, it would be useful for users not familiar with Splatter to explain what a temporal effect size of 0.4 means, roughly. Second, from the paper it's not clear how the temporal patterns are simulated. The authors only indicate that the changes are made from a baseline sample, and how the changes are passed to Splatter, but not how the changes are related across timepoints. This should be more clearly described.

**Response:** Thank you for bringing these concerns to our attention. In response to your comments, we have revised the manuscript to provide a clearer explanation of our simulation design. Specifically, we simulated three replicates/samples for each time point, and each scenario was repeated 10 times (Page 28, lines 20-22). The final power results were averaged across 10 repeats for each simulation scenario.

**Figure R1.** The temporal expression patterns across time points. We only consider the gene exihibits: (**A**) growth pattern; (**B**) recession pattern; (**C**) peak pattern; (**D**) trough pattern; (**E**) bi-plateau pattern; (**F**) multi-modal patterns.

Additionally, we acknowledge the importance of clarifying the temporal effect size for users unfamiliar with *Splatter*. Specifically, *Splatter* simulated scRNA-seq data by specifying the number of cells (using *batchCells* parameter in Splatter), and the number of time points (*group.prob*). Subsequently, we generated time point-specific effect sizes by setting the parameter *de.facloc* = 0.4, one time point at a time. Finally, we examined temporal expression patterns based on effect sizes across time points, limiting our simulations to six specific temporal expression patterns (Figure R1 or Figure S1). Other gene expression patterns were excluded in our simulations.

We have added a detailed description of our simulation design in the Material and Methods (Page 28, lines 16-22) and Results (Page 7, line 34). We believe that these additions will enhance the transparency and understanding of our simulation approach for the readers.

We appreciate your feedback, and these improvements aim to provide a more comprehensive understanding of our simulation methodology.

4. Since the power and FDR results presented are split by temporal pattern, I assume that the authors simulate explicitly the temporal patterns explicitly tested by their own model, which obviously represents a certain bias. In some contexts, especially with single-nuclei data (in which transcripts do not 'accumulate' over extended periods of time), some temporal transitions are bound to be happening between (rather than across) the timepoints, i.e. for instance having a plateau in the first few timepoints, then another plateau in the last few timepoints, without a clear gradation in-between (a 'tipping-point' change, as the authors mention in the discussion). When comparing to methods that are agnostic as to a time-response pattern, it would therefore be important to demonstrate that TDEseq's gain in power to the 4 temporal patterns modeled is not accompanied by a loss of power for other types of changes which, though different from the ones modeled, are nonetheless relevant temporal patterns. These include the two plateaus just mentioned, as well as an upregulation at a single timepoint (e.g. in differentiation datasets it's very common to have a set of genes that are different in the very first timepoint, and then stable in the others). Even if TDEseq implied a loss of power in detecting such pattern it wouldn't be a tragedy, but it would need to be discussed explicitly, in order for users to be aware of it.

**Response:** Thank you for your valuable suggestion. Indeed, we acknowledge the potential bias in our initial simulation strategy, where genes were explicitly simulated with temporal patterns such as growth, recession, peak, and trough, aligning with the patterns modeled by our method. We appreciate your insight and have taken steps to address this concern.

In response to your comments, we conducted the additional simulations introducing two different temporal expression patterns. Specifically, we simulated genes exhibiting a "bi-plateau" pattern (Figure R1E or Figure S1E), i.e., with a plateau in the first few timepoints and another in the last few timepoints; and a "multi-modal" pattern (Figure R1F or Figure S1F), i.e., with multiple modes at very first timepoint, and then stable in the others.

The results revealed that TDEseq performed well in detecting genes with a "bi-plateau" temporal expression pattern, as the flexibility of the "growth" spline function curve allows it to model such patterns effectively (Figure S8A). However, TDEseq exhibited poor performance in detecting genes with a "multi-modal" temporal expression pattern (Figure S8B). Specifically, cell-cycle-associated genes, which often exhibit periodic behavior across multiple cell cycle progressions, fall into this category, and TDEseq may struggle to detect these differentially expressed genes. We have incorporated this crucial information in the Discussion section (Page 22, lines 31 - 35) to ensure users are aware of these nuances and limitations.

Your feedback has been instrumental in enhancing the clarity and completeness of our work, and we appreciate your thoughtful comments.

5. COMPARISONS: This brings me to the comparisons. While trade-seq and impulseDE2 are relevant comparisons, there are some other options. First, the psupertime R package is supposed to detect temporal patterns related to pseudotime while simultaneously using known sample-level stages/timepoints, thus in principle being adequate for both smudged and distinct scenarios. Second, a number of reports (e.g. 10.1038/s41467-020-19894-4) indicate that pseudobulk differential expression using classical packages (e.g. edgeR) is equivalent or superior to many cell-level methods. In light of this, it would be important to compare, in the context of a simulation with replicated/multi-sample timepoints, TDEseq to a pseudobulk analysis taking the timepoints as categorical and testing dropping all of the time-related coefficients simultaneously (e.g. ~1+stage vs ~1, eventually including other relevant covariates), as is typically done in bulk RNAseq time-course analysis. This is especially important given TDEseq's main use-case being the distinct stages scenario discussed above.

**Response:** Thank you for providing valuable insights and alternative methods for comparison. We appreciate your constructive comment, and in response to your suggestion, we have expanded the comparisons in the revision as follows:

(1) For psupertime method. The psupertime is a supervised pseudotime approach to identify time-varying genes and provide better individual cell orderings (Will Macnair, et al. 2022). We performed psupertime method (<https://github.com/wmacnair/psupertime>, version 0.2.6) on simulations, finding psupertime detected a quite few number of genes (less than 30 genes; Figure R2). Furthermore, the psupertime is very time consuming for large number of cells. For example, with 3000 cells and 10000 genes, psupertime will take about 5 hours, presumably due to its primary build on penalized ordinal logistic regression models. Therefore, we excluded the psupertime in our comparison.

**Figure R2.** The comparison of temporal expression gene detection under baseline scenario (left panel) and large sample-level variation scenario (right panel). The psupertime detected 2.3% and 2.1% temporal expression genes for baseline and large sample-level variation scenarios, respectively.

(2) For pseudobulk DE methods. Both DESeq2 and edgeR are very popular differential expression analysis tools for bulk RNA-seq data. These two methods can also address the time-course RNA-seq data. We included both DESeq2 and edgeR in comparison following the previous studies (Helena L. Crowell, et al. 2020). As a result, we found TDEseq outperformed edgeR and DESeq2 in most scenarios (Figures 1 and S2-S5, S7-S9); while edgeR and DESeq2 exhibited more poweful performance in situations where the number of cells is small, expected UMI counts for each cell are large, and effect sizes are large (Figures S3D, S4C, and S5A).

We have incorporated these results in the revised manuscript to provide a more comprehensive assessment of the performance of TDEseq (Page 9, lines 6 - 11, lines 26 - 27; Page 11, lines 1 – 4; Page 16, lines 11 – 22; Page 19, lines 10 – 18). We also explicitly highlighted the scenarios where edgeR and DESeq2 may perform well and where the inherent design of TDEseq might have limitations in the Disscussion section (Page 21, lines 35 – 36; Page 23, lines 1 - 4). Your input has contributed significantly to the robustness of our evaluation, and we appreciate your thoughtful suggestions.

6. PACKAGE: The package depends on the Matrix.utils package, which was removed from CRAN a year ago. As a result, the installation fails on any decently-recent setup, and I had to find an older machine to test it. This should be solved before publication.
   - I've tested the method on a dataset of ~5k cells and 15k genes, and it took a day to finish. I understand that the package has a pseudocell capability to reduce computational load, but since the fits are independent from each other, I don't understand why no multi-threading was implemented - it's really simple to do, and makes a big difference to users.
   - In the documentation, the usage of the '@' (e.g. seurat_object@slot) to access data is not good practice and should be replaced by the accessor functions (e.g. GetAssayData and the likes). Also, in the help of the main function, the input should be more clearly defined, e.g. that 'meta' should have columns with certain names and classes (e.g. 'stage' can't be a factor but needs to be numeric).

**Response:** Thank you for providing valuable feedback, and we appreciate your efforts in testing TDEseq. We have addressed the issues you raised in the current version of the TDEseq software:

(1) Matrix.utils issue: We have removed the dependence of a repository of *Matrix.utils* to eliminate the installation problem. We have updated the software documentation (https://sqsun.github.io/software.html) to provide clearer guidance on installation of TDEseq.
(2) Multi-threading: Regarding the computational efficiency concerns, we have implemented multi-threading functionality in the TDEseq package by incorporating *doParallele* R package. Users can now take advantage of this improvement by setting the parameter *num. core* to optimize processing time (Page 7, line 20).
(3) Data structure: To promote robust coding practices, we have established an S4 class for the TDEseq object. Utilizing TDEseq has been streamlined to two key steps (https://sqsun.github.io/software.html): **1)** tde <- *CreateTDEseqObject(counts = expr_dat)* to initialize the TDEseq object, and **2)** res <- *tdeseq(object = tde)* for the actual analysis. Furthermore, TDEseq now supports various data structures, including *SingleCellExperiment* object, *Seurat* object, or gene expression matrix, etc.

These changes aim to improve user experience, resolve installation issues, and adhere to best coding practices. Your input has been instrumental in refining the TDEseq software, and we appreciate your thorough testing and constructive comments.

7. I think the average reader won't understand, from the intro, the distinction between time-resolved and time-course data. It might also not be necessary for the purpose of the paper.

**Response:** We appreciate the reviewer's observation regarding the distinction between "time-resolved" and "time-course" data in the introduction. Upon careful consideration, we acknowledge that there is no substantial difference between the two terms for the purpose of this manuscript. Therefore, we have revised the terminology to use "time-course scRNA-seq data" consistently throughout the paper, aligning it with the perspective presented in the methodology.

8. Some details in the text could be added to improve clarity, for example in the methods section, when the formula is discussed in more detailed, it would be good to spell out all variables (I had to scroll back and forth between results and methods to figure out what the variables are).

**Response:** We appreciate your feedback. To enhance clarity, we have incorporated detailed explanations of all variables in the methods section (Page 24, lines 9 - 25), ensuring that readers can easily comprehend the formula without the need to navigate back and forth between the results and methods sections.

9. In Supplementary Figure 10C, the plot doesn't at first glance convey the point made in the text (high correlation). It would be easier to interpret if it was colored by density, so that we'd see that the points not along the diagonal are a small minority. In addition, however, there's clearly a set of genes that get a much stronger linear p-value than mixed, and it would be worth discussing this briefly.
   - Check for typos (e.g. p.9 line 16, distinct)

**Response:** We appreciate the reviewer's keen observations and suggestions. In response, we conducted a thorough examination of genes exhibiting a much stronger linear *p*-value than mixed. Our analysis revealed that these genes display significant individual heterogeneity across samples, characterized by high expression in some samples but not in others. Notably, the linear version of TDEseq solely considers stage information, while the mixed version incorporates individual information, providing a more comprehensive perspective.

To address this, we have added a discussion on the advantages of the mixed version of TDEseq, supported by new results presented in Figure S14D and detailed explanations in the Results section (Page 14, lines 5 – 10) and the Discussion section (Page 21, lines 31 – 35; Page 22, lines 1 - 3). Additionally, we have replaced Figure S14C with a density plot. This modification aims to enhance the visualization, emphasizing that the majority of points align along the diagonal line.

We have also meticulously checked for typos, including the one you pointed out on page 7, line 17 ("distinct"). Your valuable feedback has significantly contributed to the refinement of our manuscript.

-------------------
**Reviewer: 2#**

**General Comments:**

This research paper introduces a method, TDEseq, which offers a non-parametric approach for detecting temporal expression patterns across multiple time points. Below, I've listed my questions and suggestions:

**Response:** Thank you very much for the constructive comments and valuable suggestions, which have substantially improved the quality of the paper. Our detailed responses are as follows:

**Specific Comments:**

1. In the introduction, the author mentioned that 'time is often treated as a categorical variable, and differential expression analysis is performed through pairwise comparisons using methods such as the two-sided Wilcoxon rank-sum test, DESeq2, or edgeR.' This statement should be refined. For example, when using edgeR, continuous variables are acceptable for constructing the model matrix.

**Response:** We appreciate the reviewer's clarification on the use of continuous variables in the context of methods like DESeq2 and edgeR. Acknowledging this, we have refined the statement in the Introduction section to better reflect the flexibility of these methods in handling continuous variables (Page 4, lines 1 and 5).

2. The color of the Tradseq legend in the supplementary figures appears to be incorrect.

**Response:** We appreciate your observation regarding the inconsistency in the color of the tradeSeq legend in the supplementary figures. We have rectified this error by updating the figures to ensure consistency between the color of the legend and the figures themselves (Figures S2-S5).

3. In the method section, it is mentioned that 'transformed genes' are used in the model to facilitate preprocessing before temporal expression gene detection. However, the term 'transformed' is not clearly explained. Is any normalization or integration method manipulation permitted? If so, then raw counts should also be allowed as well?

**Response:** We appreciate your attention to detail and acknowledge the need for clarification regarding the term 'transformed genes' in the method section.

In our approach, we performed log-normalization to transform the count nature of raw gene expression data into normalized data. While log normalization is the method specifically mentioned, we want to emphasize that other normalization methods, such as *SCTransform()*, are also acceptable within the proposed framework.

To rectify this omission, we have included this crucial information in both the Results section (Page 6, lines 10 - 11) and the Materials and Methods section (Page 24, lines 3-4), providing a more comprehensive understanding of the preprocessing steps involved.

4. Could you provide details on what the power looks like in simulation scenarios where the effect size varies?

**Response:** We appreciate your inquiry regarding the power in simulation scenarios with varying effect sizes.

In response, we have included the results that illustrate how detection power changes with different parameter settings (Figure S7), including varying effect sizes. We presented these results in the Results section (Page 9, lines 11-16). This additional visualization aims to provide a clearer understanding of the method's performance across different simulation scenarios.

5. Based on my observations, in simulations, the mixed model seems better at detecting 'growth' and 'recession,' whereas the linear model appears better at capturing the 'peak' and 'trough.' Do you have any insights into this observation?

**Response:** We appreciate the reviewer's insightful observation, and upon thorough examination of the results, we have identified a noteworthy pattern. It appears that the linear model tends to emphasize the peak/trough pattern, while the mixed model leans towards highlighting the growth/recession pattern. This phenomenon may stem from the weak peak (Figures R3E and R3F) or weak trough (Figures R3G and R3H), which were detected as growth and recession patterns by Mixed TDEseq.

**Figure R3.** Temporal expression patterns. (**A**) growth pattern; (**B**) recession pattern; (**C**) peak pattern; (**D**) trough pattern; (**E**) weak peak pattern; (**F**) weak peak pattern; (**G**) weak trough pattern; (**H**) weak trough pattern.

To provide clarity on this aspect, we have included a note in the Discussion section (Page 22, lines 34-35 and Page 23, lines 1-3) acknowledging this observation and highlighting it as a potential avenue for meaningful exploration in future research. We value your keen observations and thoughtful comments, which contribute to a more comprehensive understanding of our method's behavior.

6. The term 'batch' is not well explained in the context of simulations. From my understanding, the author simulates scenarios with varying settings for each sample. I noticed that the mixed TDEseq consistently produces well-controlled p-values and exhibits good power compared to the linear model. I believe this is due to a well-modeled relationship between expression levels. However, it may be more appropriate to avoid referring to this as a 'batch effect.'?

**Response:** We appreciate your careful consideration and thoughtful comment regarding the term 'batch' in the context of simulations. Indeed, the term ‘batch’ in our manuscript is to denote the sample-level variations. We acknowledge the potential confusion and have taken steps to address this concern.

To clarify, we have conducted a thorough examination of the manuscript and involved ‘individual heterogeneity’ or ‘sample-level variations’ to interprerate the ‘batch effects’ in simulations. Indeed, there also exists the time point-wise (i.e., stage-wise) unwanted variation, which also could be denoted as ‘batch effects’. Specifically, we denoted the stage-wise unwanted effects as batch effects, and while denoted the sample-level variations within time points as sample heterogeneity. Importantly, the random effects in the TDEseq simultaneously model aim to account for both types of effects.

We included the additional explanations thorough the manuscript (Page 9, lines 14-16; Page 21, lines 8-12; Page 28, lines 10-13), aiming to provide a clearer understanding of our simulation design and dispel any ambiguity surrounding the term 'batch.'

Thank you for your insightful comment, and we believe these additions enhance the transparency of our methodology.

7. In the discussion section, some additional details are needed following the statement 'Two versions of TDEseq were developed to accommodate different real data application scenarios.' The results of applying these new methods to real data provide valuable insights.

**Response:** We appreciate your feedback, and in response to your suggestion, we conducted a thorough examination of the results obtained from the application of the two versions of TDEseq. Notably, we found Mixed TDEseq was designed for analyzing heterogeneous samples and batch effects across different stages encountered in time-course studies, such as cancer development, while Linear TDEseq was tailored for handling smaller and heterogeneous samples utilized in time-course studies, such as cell differentiation.

The observed difference in performance is attributed to the fact that the linear version of TDEseq considers only the stage information, while the mixed version of TDEseq incorporates sample-level variations. To emphasize this advantage of the Mixed TDEseq, We have discussed in the Discussion section (Page 21, lines 32 - 36).

We appreciate your keen observations, which have contributed to a more comprehensive understanding of the strengths and limitations of the different versions of TDEseq.

**Second round of review**

**Reviewer 1**

I congratulate the authors on the great job they've done addressing my concerns, and on convincing me to use their method next time I have appropriate data.

I therefore recommend the publication of the paper, and would only have a few small requests left:

- Please include in the discussion a recap of the results on the smudged simulations, as it is important that readers make an informed choice when they suspect they are in such a scenario.

- Since there are now many lines in the plots, it might be good to use the linetype/shape to highlight the TDEseq methods

- In figures in which you plot observed vs expected -log10(pvalues), ideally include a diagonal

- Please submit the package to Bioconductor

**Reviewer 2**

The author has responded comprehensively to all my questions, offering satisfactory answers. It is encouraging that the article clearly defines the term 'batch' and compares two pseudo-bulk methods, DESeq2 and edgeR. Additionally, the inclusion of new simulations to address reviewer concerns is commendable.

However, I find the main plots added to be somewhat unclear and in need of updating for improved clarity. It would be beneficial if the author could refine these plots to ensure they are more legible and easier to interpret.

**Response to Comments from Reviewers:**

-------------------
**Reviewer: 1#**

**General Comments:**

I congratulate the authors on the great job they've done addressing my concerns, and on convincing me to use their method next time I have appropriate data.
I therefore recommend the publication of the paper, and would only have a few small requests left:
- Please include in the discussion a recap of the results on the smudged simulations, as it is important that readers make an informed choice when they suspect they are in such a scenario.
- Since there are now many lines in the plots, it might be good to use the linetype/shape to highlight the TDEseq methods
- In figures in which you plot observed vs expected -log10(pvalues), ideally include a diagonal
- Please submit the package to Bioconductor

**Response**: Thanks for your constructive requests. We have addressed those requests as follows:

(4) Smudged simulations. We have included a discussion section for the results on the smudged simulations, to guide an informed choice of TDEseq for different scenarios (Page 22, lines 4-10).
(5) Line plots. We have updated the power plots with linetype in all figures. Specifically, The TDEseq methods were highlighted using solid lines, while other methods were represented by dashed lines in the plots(Figures 1C, 1F, 2C, 3C, 4C, 4F, 5D and supplementary figures).
(6) QQ plots . We have added a diagonal line on all quantile-quantile (QQ) plots in main figures and supplementary figures(Figures 1B, 1E, 2B, 3B, 4B, 5C and supplementary figures).
(7) Bioconductor. We have now submitted TDEseq to Bioconductor. The submission is currently pending review by the administrator.

**Reviewer: 2#**

**General Comments:**

The author has responded comprehensively to all my questions, offering satisfactory answers. It is encouraging that the article clearly defines the term 'batch' and compares two pseudo-bulk methods, DESeq2 and edgeR. Additionally, the inclusion of new simulations to address reviewer concerns is commendable.

However, I find the main plots added to be somewhat unclear and in need of updating for improved clarity. It would be beneficial if the author could refine these plots to ensure they are more legible and easier to interpret.

**Response**: Thanks for your comments. We have updated all line plots with shapes to highlight the TDEseq methods, i.e., the TDEseq methods were highlighted using solid lines, while other methods were represented by dashed lines in the plots(Figures 1C, 1F, 2C, 3C, 4C, 4F, 5D and supplementary figures).
